# LncRNA LINC01857 drives pancreatic adenocarcinoma progression via modulating miR-19a-3p/SMOC2

**DOI:** 10.1016/j.clinsp.2022.100047

**Published:** 2022-06-02

**Authors:** Yeting Lu, Dongjian Ying, Yuan Tian, Yi Ruan, Gong Cheng, Kaiji Lv, Xinhua Zhou, Shuo Han

**Affiliations:** a Department of General Surgery, The Affiliated Lihuili Hospital, Ningbo University(Ningbo Medical Center Lihuili Hospital), Ningbo 315100, Zhejiang, China.; b Department of Healthcare Security and Price Management, The Affiliated Lihuili Hospital, Ningbo University (Ningbo Medical Center Lihuili Hospital), Ningbo 315100, Zhejiang, China

**Keywords:** LINC01857, miR-19a-3p, SMOC2, Pancreatic adenocarcinoma

## Abstract

**Objectives:**

Emerging evidence has demonstrated that LINC01857 exerts a pivotal function in many cancers. However, its function in Pancreatic Ductal Adenocarcinoma (PDAC) still remains unclear. This study was designed to investigate the regulatory character of LINC01857 in PDAC.

**Methods:**

Bioinformatic tools and databases were used to seek potential miRNAs and mRNAs. Gene expression was evaluated by Reverse Transcription quantitative real-time Polymerase Chain Reaction (RT-qPCR), and western blot was used for protein level detection. A subcellular fraction assay was done to ascertain the location of LINC01857 in PANC-1 and BxPC-3 human pancreatic cancer cells. CCK-8, EdU, wound healing and Transwell assays were performed to inquire into the influence of LINC01857, and SPARC -related Modular Calcium-binding protein-2 (SMOC2) on cell viability, proliferation, migration, and invasion, respectively. The interaction between LINC01857 and its downstream genes was explored by RNA immunoprecipitation and luciferase reporter assays.

**Results:**

LINC01857 levels were significantly elevated in PDAC. Knockdown of LINC01857 significantly restrained the proliferation, migration, invasion, and Epithelial-Mesenchymal Transition (EMT) process of PDAC cells. MiR-19a-3p was a downstream target of LINC01857, and miR-19a-3p levels were significantly decreased in PDAC cells. In addition, SMOC2 expression had a negative correlation with that of miR-19a-3p, and SMOC2 was a downstream target of miR-19a-3p. Furthermore, SMOC2 upregulation partially abolished the inhibitive influence of LINC01857 downregulation on cell proliferation, migration, invasion, and the EMT process.

**Conclusion:**

LINC01857 promotes malignant phenotypes of PDAC cells via upregulation of SMOC2 by interacting with miR-19a-3p.

## Highlights

• LINC01857 is upregulated in PAAD and promotes malignant cellular behaviors.
• LINC01857 interacts with miR-19a-3p to regulate SMOC2 expression.
• LINC01857 promotes malignant cellular phenotypes by upregulating SMOC2.

**Abbreviations**
PAAD: Pancreatic Adenocarcinoma
EMT: Epithelial-Mesenchymal Transition
lncRNAs: Long noncoding RNAs
PVT1: lncRNA Pvt1 oncogene
THAP9-AS1: lncRNA THAP9 antisense RNA 1
MAP4K4: Mitogen-Activated Protein Kinase kinase kinase kinase 4

## Introduction

Pancreatic Ductal Adenocarcinoma (PDAC) is a fatal malignancy that accounts for 2% of all cancers and 5% of cancer-related deaths.^1^ It is so aggressive that fewer than four percent of PDAC patients will survive five years beyond the diagnosis.^1^ Risk factors of PDAC are identified as smoking, obesity, diabetes, diet and inactivity, but the exact causes are still ambiguous.^2^^,^^3^ Until now, surgical resection remains the most effective method for PDAC, although it is only adapted for a few eligible patients.^4^^,^^5^ Therefore, it is of great clinical significance to conduct in-depth research on the molecular alterations of PDAC and to explore new treatment methods.

Long noncoding RNAs (lncRNAs) are a group of transcribed RNAs containing over 200 nucleotides.^6^ Many studies have shown that lncRNAs exert critical function on pancreatic adenocarcinoma. For example, lncRNA Pvt1 oncogene (PVT1) was reported to promote pancreatic adenocarcinoma progression via the miR-20a-5p/ULK1 axis, which is associated with poor prognosis.^7^ lncRNA THAP9 antisense RNA 1 (THAP9-AS1) promotes the growth of pancreatic ductal adenocarcinoma and causes an unfavorable clinical outcome by binding to miR-484.^8^ To date, as a novelty identified lncRNA, LINC01857 has been suggested to promote the malignant development of several cancers. For example, it was reported to encourage tumorigenesis and metastasis of glioma by regulating the miR-1281/TRIM65 axis.^9^ LINC01857 is also confirmed to be upregulated in gastric cancer tissues and related to lymph node metastasis and unfavorable outcome of patients with gastric cancer.^10^ Nevertheless, the biological character and the mechanism of LINC01857 in PDAC have not been explored.

Importantly, increasing evidence has demonstrated that lncRNAs may regulate the levels of downstream targets by binding to miRNAs, serving as competing endogenous RNAs (ceRNAs) to alter mRNA expression.^11^, ^12^, ^13^ For example, lncRNA DLEU2L is suggested to inhibit gemcitabine resistance in PDAC cells by functioning as a sponge for miR-210-3p.^14^ LINC00857 was previously proved to be associated with PDAC progression by competitively binding to miR-340-5p, leading to an increase in TGFA levels.^15^ Additionally, LINC01857 has been reported to exert the same biological effect by competitively interacting with miRNAs. For example, it has been confirmed to promote cell proliferation but inhibit apoptosis of diffuse large B-cell lymphoma cells. It competitively binds to miR-141-3p which can target Mitogen-Activated Protein Kinase Kinase Kinase Kinase 4 (MAP4K4) to influence cancer progression.^16^ Here, the authors hypothesize that LINC01857 and miR-19a-3p may have regulatory roles in PDAC development.

Additionally, Secreted Protein Acidic and Rich in Cysteine (SPARC)-related Modular Calcium-binding protein 2 (SMOC2) is predicted as a target gene of miR-19a-3p. SMOC2 is a protein that stimulates endothelial cell migration and proliferation.^17^ SMOC2 was also identified to exert a regulatory function on various cancers and diseases,^18^, ^19^, ^20^, ^21^ including pancreatic cancer.^22^ However, the role and mechanism of LINC01857/ miR-19a-3p/ SMOC2 axis in PDAC development have not been studied yet.

In conclusion, the principal objective of the present study was to research the biological character and underlying mechanism of lncRNA LINC01857 in pancreatic adenocarcinoma. The study may provide an innovative strategy for PDAC treatment.

## Materials and methods

### Bioinformatics analyses

The level of LINC01857 and SMOC2 in PDAC tissues was assessed through GEPIA (http://gepia.cancer-pku.cn/). The starBase website (http://www.sysu.edu.cn/) was applied for predicting the downstream miRNAs of LINC01857, and three miRNAs (miR-19a-3p, miR-4731-5p and miR-19b-3p) were identified. The target genes of miR-19a-3p were also predicted using the starBase with the predicted program of miRmap, RNA22, and TargetScan, and three genes (SMOC2, ATP6V1B2 and UBAP2L) were identified.

### Tissue samples and cell lines

The PDAC tissues (n = 78) and adjacent normal samples (n = 78) were acquired from PDAC patients at the Affiliated Lihuili Hospital, Ningbo University. All the specimens were instantly reposited at -80°C. All patients had not received other anticancer treatment prior to operation and signed the informed consent of this study before surgery. This investigation was approved by the Ethics Committee of the Affiliated Lihuili Hospital, Ningbo University.

The PDAC cells (PANC-1, BxPC-3 and AsPC-1) and Human normal Pancreatic Ductal Epithelial cell line (HPED) were purchased from China Cell Culture Center (Shanghai, China). The cells were incubated using RPMI-1640 medium (Invitrogen, Carlsbad, CA, USA) that contains 10% fetal bovine serum (Hyclone, USA) and 1% PenStrep (100 U/mL penicillium and 100 μg/mL Streptomycin) in a humid environment with 5% CO_2_ at 37°C.

### Cell transfection

To silence LINC01857, PANC-1 and BxPC-3 cells were transfected with short hairpin RNA (sh-RNA) targeting LINC01857 (sh-LINC01857#1/2) or the corresponding negative control sh-NC (GenePharma, Shanghai, China) for 48h. To upregulate miR-19a-3p, miR-19a-3p mimics (Biomics Inc., DE, USA) and NC mimics (Biomics Inc.) were transfected into PANC-1 and BxPC-3 cells for 48h. Stable transfections were conducted with Lipofectamine 2000 reagent (Invitrogen). For overexpression of SMOC2, PANC-1 and BxPC-3 cells were transfected with pcDNA3.1/SMOC2 or control pcDNA3.1 vectors (GenePharma). The transfection efficiency was analyzed by RT-qPCR after 48h.

### Real-time reverse-transcription polymerase chain reaction (RT-qPCR)

Total RNA was extracted from PDAC cells and tissues with a TRIzol reagent (Takara Bio Inc., Tokyo, Japan) following the manufacturer's recommendations and was reverse transcribed into first-strand cDNA with a cDNA Reverse Transcription Kit (T Merck KGaA, Darmstadt, German). A TB Green Premix Ex Taq II reagent kit (RR820B, Takara) was implemented to conduct RT-qPCR using an ABI7500 real-time qPCR system (Thermo Fisher Scientific, Waltham, MA, USA). GAPDH was considered to be the introcontrol for LINC01857, SMOC2 and candidate miRNAs. U6 was considered to be the internal control for miRNAs. The relative expression level of LINC01857, SMOC2, and miR-19a-3p was measured by the 2^−ΔΔCt^ method.^23^ Primer sequences applied in PCR have been shown in Table 1.

**Table 1:** Primer sequences of RT-Qpcr.

Target		Primer sequence (5’‒3’)
LINC01857	Forward	CAGCCTTCGGAACTATGGA
	Reverse	GCGGAAACTGTTAGATGCA
miR-19a-3p	Forward	GGCGGGGAAAGTGTGTCT
	Reverse	GTGCAGTCGTGGCGTGTG
miR-19b-3p	Forward	CGTGTGCAAATCCATGCAA
	Reverse	GTCGTATCCAGTGCAGGGTCCGAGGTATTCGCACTGGATACGACTCAGTT
miR-4731-5p	Forward	GGGGGCCACATGAGT
	Reverse	GGTCCAGTTTTTTTTTTTTTTTCACA
ATP6V1B2	Forward	TAGTTCAGGTATTTGAAGGGAC
	Reverse	GGTGTTCGGAGAATATCCC
UBAP2L	Forward	TTTCCCACACCCACTACTC
	Reverse	GAACTTTGTGAGGTCACCAG
SMOC2	Forward	TACAAGAACTGATGGGCTG
	Reverse	TTTCCTTGGCTGTCTATTAGAC
GAPDH	Forward	GGAGCGAGATCCCTCCAAAAT
	Reverse	GGCTGTTGTCATACTTCTCATGG
U6	Forward	CTCGCTTCGGCAGCACA
	Reverse	AACGCTTCACGAATTTGCGT

### 5-Ethynyl-2′-deoxyuridine (EdU) assay

A 5-Ethynyl-2-Deoxyuridine (EdU) labeling kit (KeyGEN, Nanjing, China) was conducted to evaluate cell proliferation in accord with the manufacturer's recommendations. After transfection, PANC-1 and BxPC-3 cell lines were grown in 96-well plates at 5 × 10^3^ cells/well for 48h. Afterward, 50 μM EdU labeling media (KeyGEN) was added to the plates, and the cell lines were cultured with 5% CO_2_ at 37°C for 2h. After being fixed with 4% paraformaldehyde containing Phosphate Buffered Saline (PBS), the cells were stained with an anti-EdU working solution. Cell nuclei were labeled using DAPI. The EdU-positive cells were counted with fluorescent microscopy (KEYENCE, Osaka, Japan). The experiment was conducted three times.

### Western blot

Cell lysates from PANC-1 and BxPC-3 cell lines were collected by RIPA buffer with protease inhibitors. Protein concentration was evaluated using a BCA kit. Then, 20 μg of protein sample was isolated by 10% SDS-PAGE and transferred onto a PVDF membrane. Next, the membrane was clogged with 5% defatted milk powder, washed with PBS once, and then cultured overnight at 4°C with the primary antibodies as follow: rabbit anti-E cadherin (ab40772, 1:10000, Abcam, Shanghai, China), rabbit anti-N cadherin (ab76011, 1:5000, Abcam), rabbit anti-vimentin (ab137321, 1:500, Abcam), rabbit anti-SMOC2 (PA5-31892, 1:1000, Invitrogen), anti-GAPDH (ab9485, 1:2500, Abcam). After being washed using PBS at room temperature 3 times (5 min each time), the membrane was cultured with secondary antibody goat anti-rabbit IgG H&L (ab150077, 1:200, Abcam) at 37°C for 1h. After being rinsed thrice with PBS, the membrane was immersed in a reinforced chemiluminescence reaction solution for 1 min and quantified using Image Lab™ Software (Bio-Rad, USA). The experiment was repeated 3 times.

### Luciferase reporter assay

To probe the interaction between LINC01857 and miR-19a-3p, wide type and mutant miR-19a-3p were cloned into the firefly luciferase gene reporter vector and then were co-transfected with sh-LINC01857 and sh-NC into PANC-1 and BxPC-3 cell lines. To explore the interaction between miR-19a-3p and SMOC2, wide type or mutant 3’UTR of SMOC2 were cloned into the luciferase reporter vector and then were cotransfected with miR-19a-3p mimics and NC mimics into PANC-1 and BxPC-3 cells. A Dual-Luciferase® Reporter Assay System kit (Promega, Beijing, China) was implemented to assess luciferase activity.

### RNA immunoprecipitation (RIP) assay

To assess the mutual effect among LINC01857, miR-19a-3p, and SMOC2, a RIP kit (Millipore) was used in accord with the manufacturer's protocols. All the cell lysates from PANC-1 and BxPC-3 cell lines were incubated in RIP immunoprecipitation buffer harboring magnetic beads conjugated with Ago2 antibody. IgG was considered a negative control. After being cultured at 4°C for 2h, the RNA coprecipitation was extracted from the beads and measured by RT-qPCR. The experiment was conducted in triplicate.

### CCK-8 assay

CCK-8 assay was performed to evaluate cell viability. After transfection, PANC-1 and BxPC-3 cell lines were plated into 96-well plates (1 × 10^4^ cells/well). At 24h, 48h, and 72h, the Cell Counting Kit-8 (CCK-8; Glpbio, Montclair, CA, USA) reagent was added. Then, the cells were incubated for 2h at 37°C, and a microplate reader (Bio-Tek Instruments, USA) was applied to measure absorbance at 450 nm. All experiments were conducted three times independently.

### Subcellular fractionation assay

Nuclear, cytoplasmic, and total RNA was separated from the cancer cells with a PARIS™ kit (Thermo Fisher Scientific). The PANC-1 and BxPC-3 cell lines were seeded into a 10-cm Petri dish and collected following the manufacturer's protocols. After being washed with PBS, the cells were resuspended with 300 μL ice-cold cell fractionation buffer and cultured on ice for 5‒10 min. Later, the cells were centrifuged at 500 × g, 4°C, for 3 min. Next, the cytoplasmic fraction was isolated from the nuclear pellet. The subcellular distribution of LINC01857 was analyzed by RT-qPCR. U6 and GAPDH were regarded as nuclear and cytoplasmic controls, respectively.

### Transwell assay

Cell invasion in PDAC was measured with Transwell chambers (Corning Inc., Corning, NY, USA). The chambers were pre-coated with Matrigel (BD Biosciences). The cells were collected after being transfected for 48h, then rinsed with PBS and resuspended in DMEM without FBS. The upper chambers were added with 100 μL of suspension that contains 5 × 10^4^ cells. The Transwell chambers were put into a 24-well plate covered with 500 μL of DMEM harboring 10% of FBS. After being inoculated for 24h, the invasive cells were fixed with 100% methanol and dyed with 0.5% crystal violet. After being rinsed with PBS, the cells were imaged by an inverted microscope (Experimental Reagents Co., Ltd, Shanghai, China). The invasion of cells was assessed by counting the invaded cells. Five fields of view were taken to count. The experiment was conducted in triplicate.

### Wound healing assay

The migration of PANC-1 and BxPC-3 cell lines was investigated by wound healing assays. Each cell monolayer was scraped in straight lines with a 10 μL pipette tip and rinsed twice using PBS when the confluence of the transfected cells increased to 80%. The cells were incubated in a medium containing 3% FBS and 1% penicillin (KeyGEN, Nanjing, China). Images were taken at 0 and 24h after the initial scratch. was used to calculate cell wound healing rate was calculated using Image J (National Institutes of Health) software. The calculation formula is as follows: (the original wound areas – the actual wound areas at different times) / (the original wound areas). The experiment was conducted in triplicate.

### Statistical analysis

Experimental assays were repeated three times and the data are presented as the mean ± standard deviation. Comparisons were made by Student's *t*-tests or one-way analysis of variance (ANOVA) followed by Tukey's posthoc analysis. Expression correlations between genes in PDAC tissues were evaluated by Spearman's correlation analysis. The value of *p* < 0.05 was deemed to have statistical significance.

## Results

### Expression of LINC01857 is elevated in PDAC tissues and cells

**Fig. 1:**
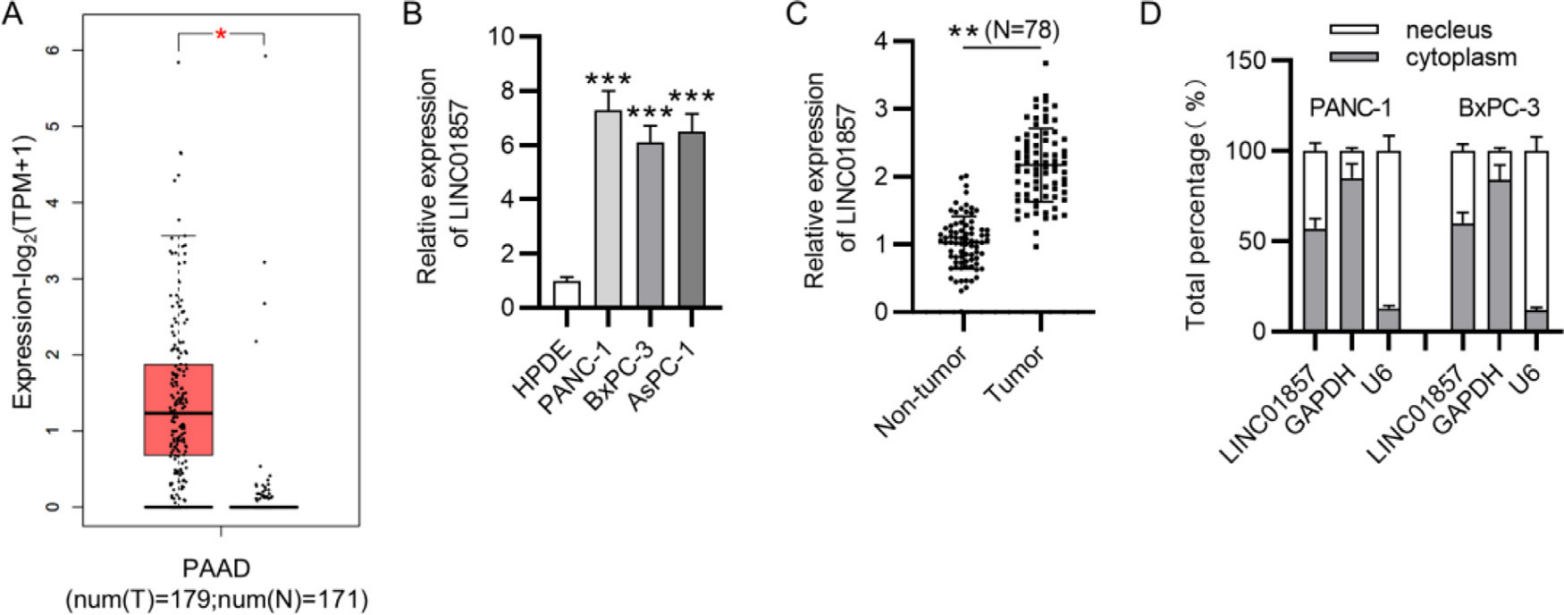
LINC01857 is highly expressed in PDAC and is mainly located in cytoplasm. (A) LINC01857 expression levels in 179 tumor samples and 171 normal ones were shown by GEPIA (http://gepia.cancer-pku.cn/). (B) LINC01857 levels in HPDE, PANC-1, BxPC-3 and AsPC-1 cells were analyzed by RT-qPCR. (C) The levels of LINC01857 in 78 tumor tissues and pair-matched non-tumor tissues was analyzed by RT-qPCR. (D) LINC01857 location was confirmed by subcellular fraction assays. *p < 0.05, ^⁎⁎^*p <* 0.01, ^⁎⁎⁎^*p <* 0.001.

The GEPIA database revealed that LINC01857 levels were markedly upregulated in 179 tumor samples in comparison with that in 171 normal ones (Fig. 1A). In addition, the RT-qPCR result displayed that LINC01857 expression in PDAC cells (PANC-1, BxPC-3 and AsPC-1) was higher than in normal cells (HPDE) (Fig. 1B). The authors subsequently found that LINC01857 levels were elevated in 78 tumor samples, compared with that in 78 pair-matched normal ones (Fig. 1C). Further, to confirm the exact location of LINC01857, a subcellular fraction assay and the RT-qPCR analysis was conducted, and the result elucidated that LINC01857 was mainly located in the cytoplasm of PANC-1 and BxPC-3 cell lines (Fig. 1D). In summary, the results above suggested that LINC01857 was highly expressed in PDAC and was mainly located in the cytoplasm.

### LINC01857 knockdown inhibits PDAC cell proliferation, migration and invasion

**Fig. 2:**
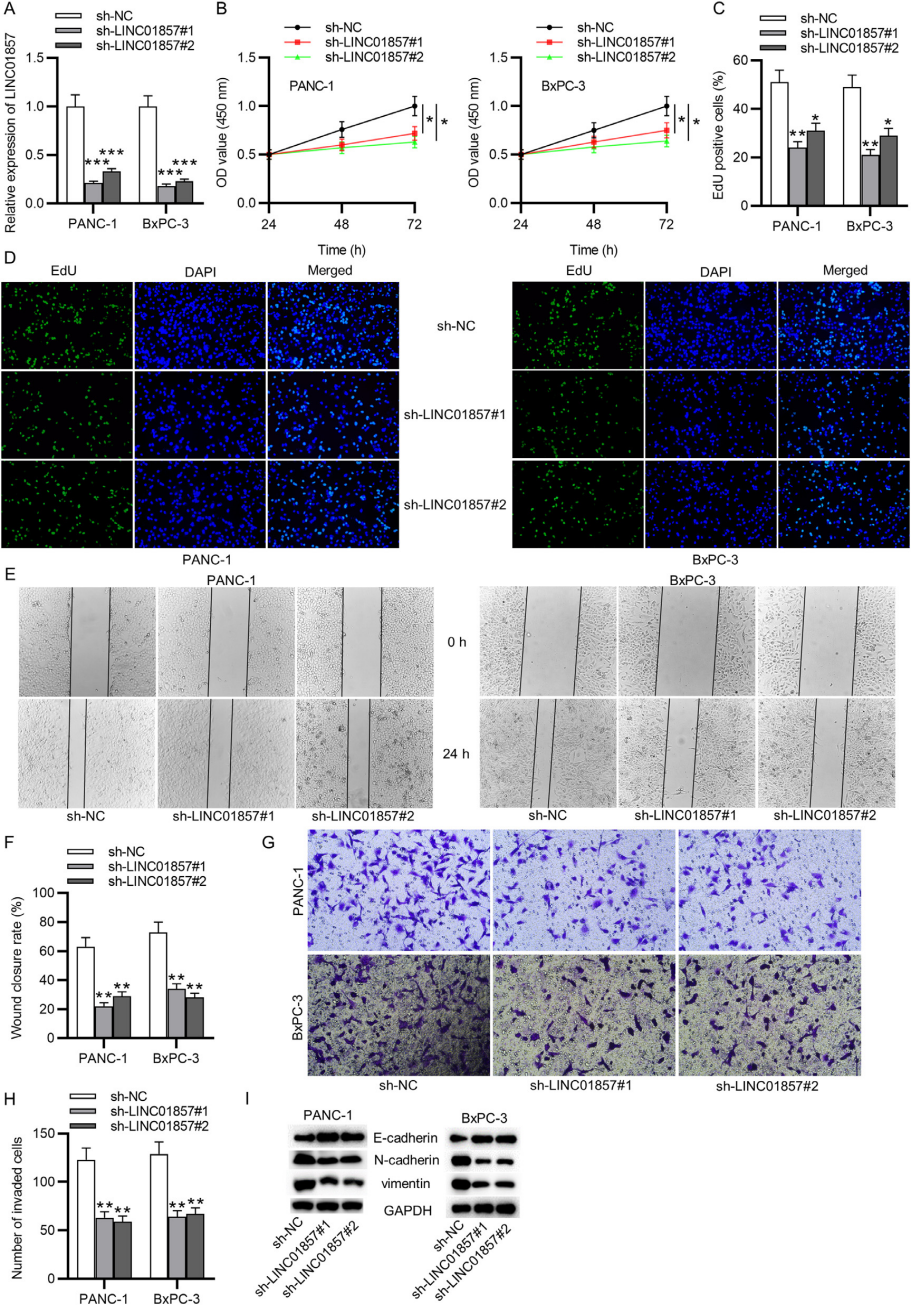
LINC01857 downregulation inhibits cell proliferation, migration, and invasion. (A) The transfection efficiency of sh-LINC01857#1/2 was tested by RT-qPCR analysis. (B) The viability of PANC-1 and BxPC-3 cells transfected with sh-LINC01857#1/2 was measured by CCK-8 assays. (C‒D) The proliferation of PANC-1 and BxPC-3 cells with the above transfection was detected by EdU assays. (E‒F) Cell migration was analyzed after transfection by wound healing assays. (G‒H) Transwell assays were conducted to analyze the invasion in PANC-1 and BxPC-3 cell lines. (I) The protein level of E-cadherin, N-cadherin and vimentin in cells with the above transfection was assessed by western blot. *p < 0.05, ^⁎⁎^*p <* 0.01, ^⁎⁎⁎^*p <* 0.001.

PANC-1 cells and BxPC-3 cells were transfected with sh-LINC01857#1/2 to assess the effects of LINC01857 on PDAC cellular processes. The RT-qPCR analysis result showed that LINC01857 levels in PANC-1 and BxPC-3 cell lines were markedly reduced by sh-LINC01857#1/2 compared with the control group (Fig. 2A). Subsequently, CCK-8 assays elucidated that sh-LINC01857#1/2 restrained the viability of PANC-1 and BxPC-3 cells markedly in comparison with the sh-NC group (Fig. 2B). Additionally, the EdU assay illustrated that cell proliferation in PDAC was suppressed by silenced LINC01857 (Fig. 2C‒D), indicating that LNCK01857 knockdown repressed the proliferation of PDAC cells. Furthermore, wound healing assays were done to examine the influence of LINC01857 on PDAC cell migration. The result manifested that the wound closure rate of PANC-1 and BxPC-3 cell lines was greatly decreased by LINC01857 knockdown in comparison with the control group, and the wound closure rate of the cancer cells was markedly decreased by LINC01857 downregulation (Fig. 2E‒F), indicating that LINC01857 knockdown repressed PDAC cell migration. In addition, according to Transwell assays, the invaded cancer cells were greatly reduced after transfection with sh-LINC01857#1/2 in PANC-1 cells and BxPC-3 cells (Fig. 2G‒H). Furthermore, the protein level of E-cadherin was raised by LINC01857 knockdown while that of N-cadherin and vimentin was markedly decreased in PANC-1 and BxPC-3 cells, as measured by western blot (Fig 2I). In summary, these results elucidated that LINC01857 downregulation restrains cell proliferation, migration, invasion, and EMT process in pancreatic carcinoma.

### LINC01857 binds to miR-19a-3p

**Fig. 3:**
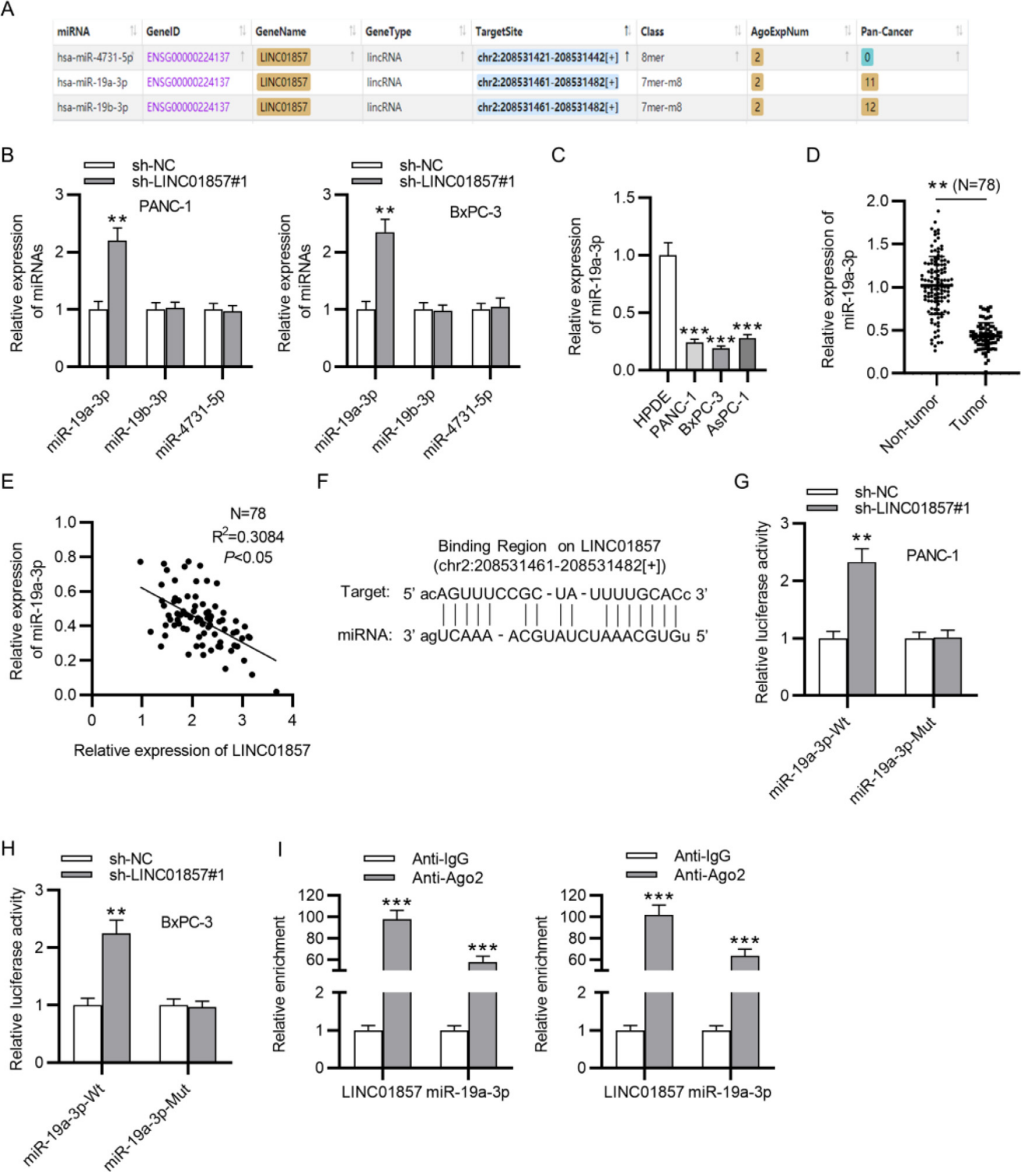
LINC01857 can bind to miR-19a-3p. (A) Potential miRNAs harboring binding site with LINC01857 were shown by the starBase database (http://www.sysu.edu.cn/). (B) The influence of LINC01857 knockdown on the expression levels of candidate miRNAs was analyzed by RT-qPCR analysis. (C) RT-qPCR was performed to analyze miR-19a-3p levels in HPDE, PANC-1, BxPC-3 and AsPC-1 cells. (D) MiR-19a-3p level in 78 tumor tissues and 78 pair-matched non-tumor tissues was analyzed with RT-qPCR. (E) The relationship between the levels of LINC01857 and those of miR-19a-3p in PDAC were investigated by Spearman's correlation analysis. (F) The binding site between LINC01857 and miR-19a-3p was shown by the starBase (http://www.sysu.edu.cn/). (G‒H) A luciferase reporter assay was done to affirm the interaction between miR-19a-3p and LINC01857. (I) The RIP assay was conducted to examine whether LINC01857 and miR-19a-3p coexist in RISCs. ^⁎⁎^*p <* 0.01, ^⁎⁎⁎^*p <* 0.001.

The mechanism by which LINC01857 modulates PDAC progression was then investigated. The starBase website shows that miR-4731-5p, miR-19b-3p and miR-19a-3p contain binding sites with LINC01857 (Fig. 3A). However, the RT-qPCR analysis indicated that LINC01857 knockdown only significantly raised miR-19a-3p levels in PANC-1 and BxPC-3 cell lines while exerting an inconspicuous effect on the levels of miR-19b-3p and miR-4731-5p (Fig. 3B) Therefore, miR-19a-3p was chosen for future study. Then, miR-19a-3p levels in HPDE, PANC-1, BxPC-3, and AsPC-1 cells were assessed by RT-qPCR analysis which elucidated that miR-19a-3p expression was knocked down in cancer cells (PANC-1, BxPC-3, AsPC-1) in comparison with that in the normal cells (HPDE) (Fig. 3C). Further, miR-19a-3p levels in 78 tumor samples were downregulated markedly compared with that in pair-matched normal ones, as determined by RT-qPCR analysis (Fig. 3D). According to Spearman's correlation analysis, LINC01857 expression had a negative correlation with that of miR-19a-3p in PDAC (Fig. 3E). The binding site between LINC01857 and miR-19a-3p was predicted by the starBase (Fig. 3F). To investigate the binding capability of LINC01857 and miR-19a-3p, a luciferase reporter assay was conducted. The result revealed that the luciferase activity of wide-type miR-19a-3p was enhanced by LINC01857 knockdown while that of mutant miR-19a-3p was not markedly changed in PDAC cells (Fig. 3G‒H). In addition, the RIP assay result presented that both LINC01857 and miR-19a-3p were markedly enriched in anti-Ago2 immunoprecipitation compared with the control group, indicating that they coexisted in RNA Induced Silencing Complex (RISC) (Fig. 3I). These results all suggested that LINC01857 binds to miR-19a-3p in PDAC.

### SMOC2 is a target of miR-19a-3p

**Fig. 4:**
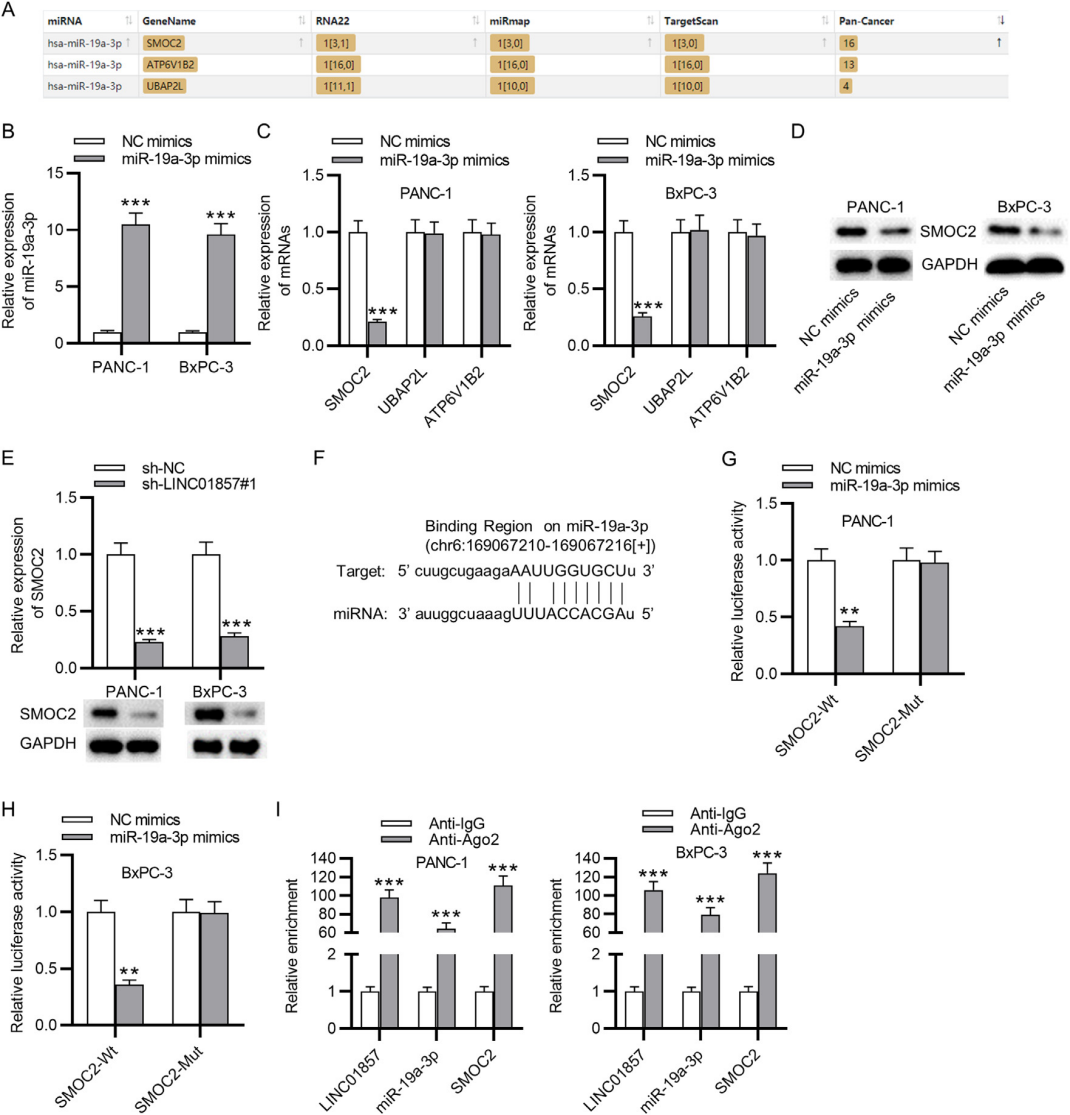
MiR-19a-3p targets SMOC2. (A) Potential mRNAs that can bind with miR-19a-3p were predicted by the starBase (miRmap, RNA22 and Targetscan, http://www.sysu.edu.cn/). (B) RT-qPCR was performed for assessing the expression efficiency of miR-19a-3p in cancer cells. (C) The levels of candidate mRNAs affected by miR-19a-3p overexpression were assessed by RT-qPCR. (D) Western blot was done to evaluate the influence of miR-19a-3p overexpression on the protein level of SMOC2. (E) The effect of LINC01857 downregulation on SMOC2 expression was explored by RT-qPCR and western blot. (F) The binding site between miR-19a-3p and SMOC2 was displayed by Targetscan software (http://www.sysu.edu.cn/). (G‒H) A luciferase reporter assay was performed to validate of the interaction between SMOC2 and miR-19a-3p. (I) The RIP assay was used to test whether LINC01857, SMOC2 and miR-19a-3p coexist in RISCs. ^⁎⁎^*p <* 0.01, ^⁎⁎⁎^*p <* 0.001.

To Figure out the downstream target of miR-19a-3p, the starBase databases (miRmap, RNA22, Targetscan) were used to predict potential mRNAs and the result showed that SMOC2, ATP6V182 and UBAP2L potentially harbor binding sites with miR-19a-3p (Fig. 4A). MiR-19a-3p mimics were transfected into PANC-1 and BxPC-3 cells, and then as shown by RT-qPCR, the levels of miR-19a-3p expression in the cancer cells were significantly upregulated compared with the control group, indicating the successful transfection (Fig. 4B). Next, in comparison with the NC-mimics group, the level of SMOC2 in PANC-1 and BxPC-3 cells were significantly decreased by miR-19a-3p downregulation while the levels of ATP6V1B2 and UBAP2L were nearly unchanged, as determined by RT-qPCR (Fig. 4C). Thus, SOMC2 was selected for further study. Western blot analysis results revealed that miR-19a-3p upregulation significantly restrained the protein levels of SMOC2 in PDAC cells (Fig. 4D), and SMOC2 expression levels and protein levels in PDAC cells were decreased by LINC01857 knockdown significantly, as shown by RT-qPCR and western blot analyses (Fig. 4E). These results suggested that SMOC2 may interact with LINC01857 and miR-19a-3p to influence PDAC progression. According to the software Targetscan, the binding site between SOMC2 and miR-19a-3p was predicted (Fig. 4F). A luciferase reporter assay result revealed that the luciferase activity of SMOC2-Wt in the cancer cell lines was markedly reduced by miR-19a-3p upregulation, while that of SMOC2-Mut was not significantly changed, suggesting that miR-19a-3p directly targeted SOMC2 (Fig. 4G‒H). Subsequently, the RIP assay was performed, showing that LINC01857, miR-19a-3p, and SOMC2 were all enriched in the precipitation with Ago2 antibody, in comparison with those in the anti-IgG group (Fig. 4I). In conclusion, the result above elucidated that miR-19a-3p targets SMOC2 in PDAC.

### SMOC2 is overexpressed in PDAC tissues and cancer cells

**Fig. 5:**
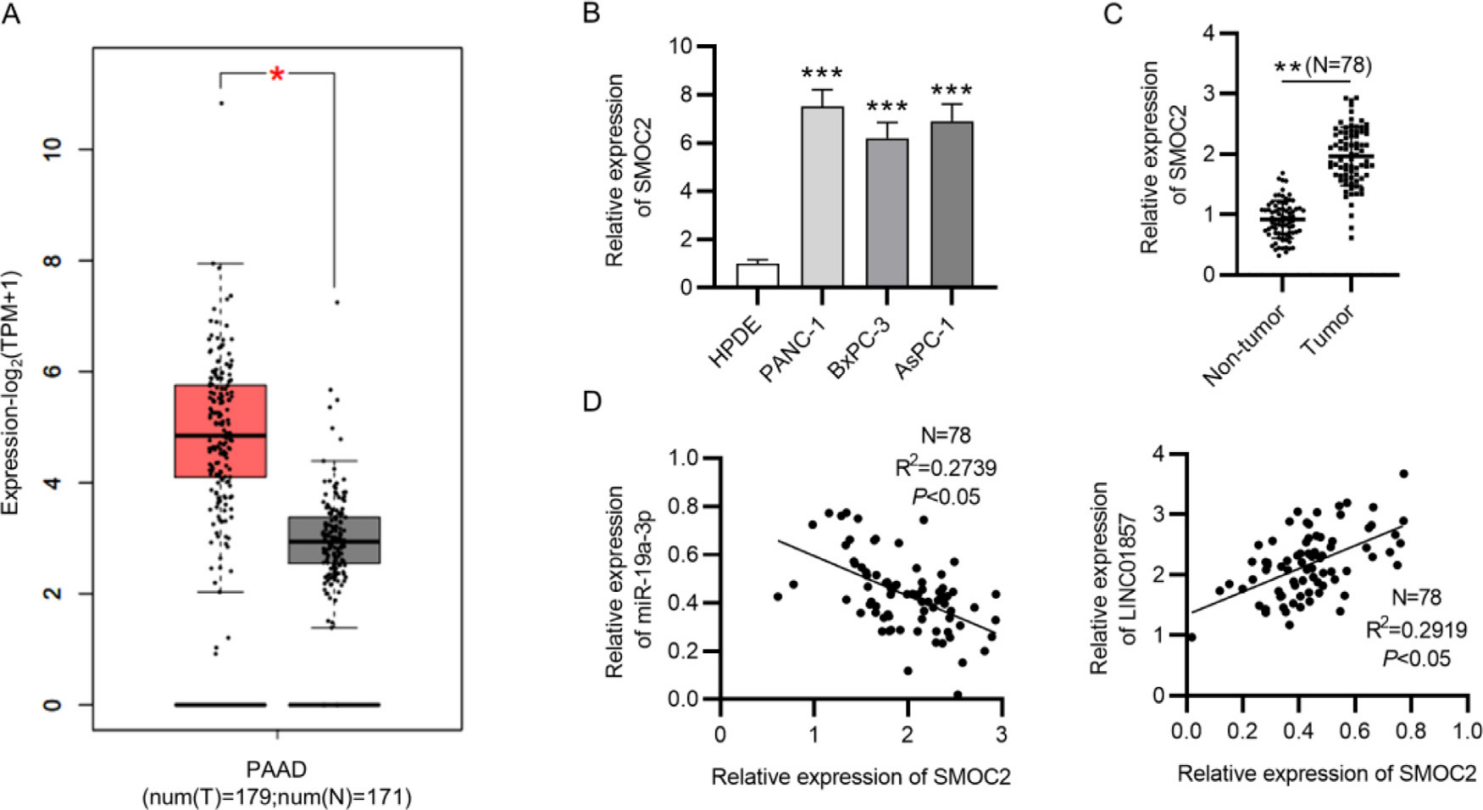
SMOC2 is upregulated in PDAC tissues. (A) GEPIA database showed SMOC2 levels in 179 tumor tissues and 171 normal tissues (http://gepia.cancer-pku.cn/). (B) SMOC2 levels in HPDE, PANC-1, AsPC-1and BxPC-3 cells were analyzed by RT-qPCR. (C) SMOC2 levels in 78 tumor samples and 78 pair-matched normal samples was determined by RT-qPCR. (D) Spearman's correlation analysis was implemented for the analysis of the correlation between SMOC2 and LINC01857 and that between SMOC2 and miR-19a-3p in PDAC tissues. *p < 0.05, ^⁎⁎^*p <* 0.01, ^⁎⁎⁎^*p <* 0.001.

As displayed by the GEPIA database, the levels of SMOC2 were markedly higher in 179 PDAC tumor tissues than in 171 normal samples (Fig. 5A). Then, RT-qPCR analysis indicated that the SMOC2 level was markedly increased in PANC-1, BxPC-3, and AsPC-1 cells in comparison with that in the HPDE cell line (Fig. 5B). The level of SMOC2 in 78 tumor samples was markedly higher than in pair-matched nontumor tissues (Fig. 5C). In addition, according to Spearman's correlation analysis, SMOC2 levels were confirmed to have a negative correlation with miR-19a-3p expression while having a positive correlation with the expression of LINC01857 in PDAC tissues (Fig. 5D). These results suggested that SMOC2 was significantly overexpressed in PDAC tissues and cells.

### SMOC2 overexpression reverses the suppressive influence of LINC01857 downregulation on cell proliferation, migration, and invasion

**Fig. 6:**
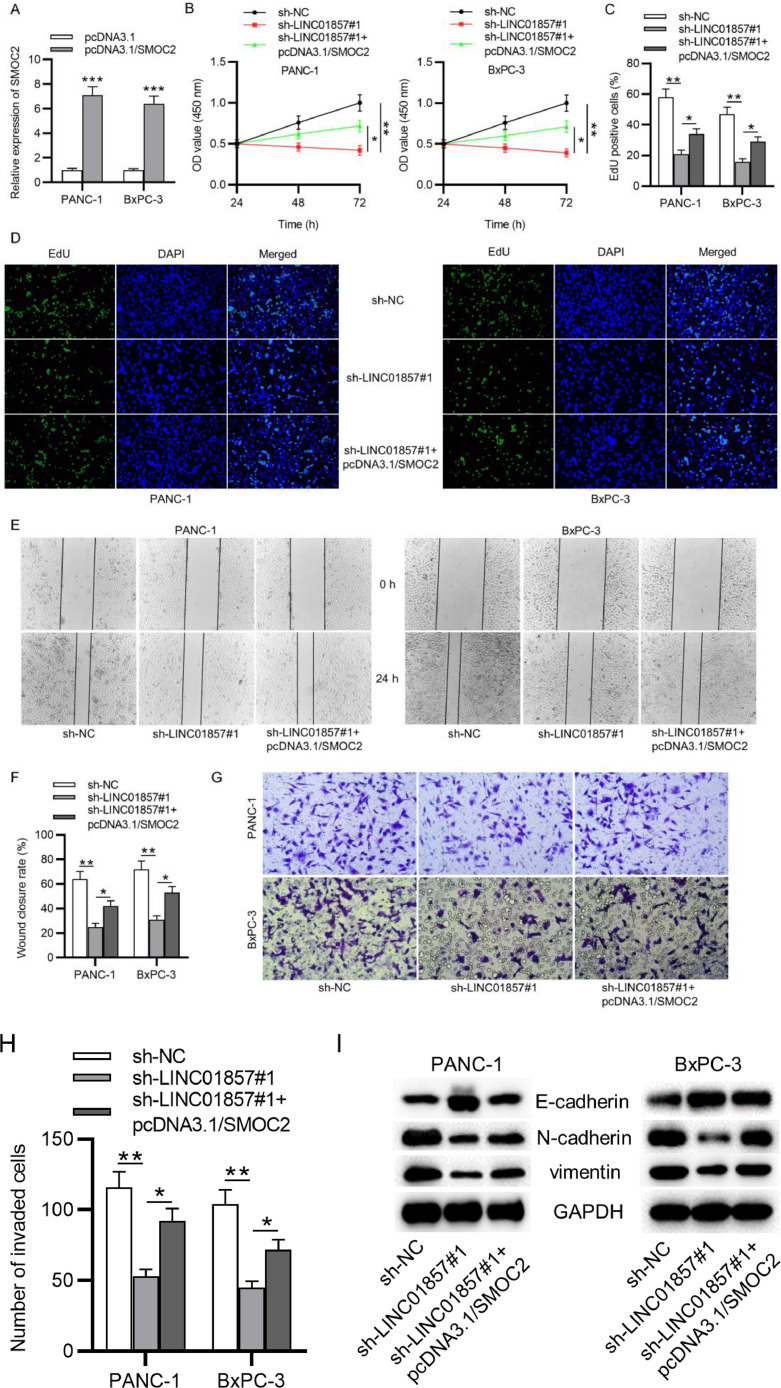
SMOC2 overexpression reverses the influence of LINC01857 downregulation on cell proliferation, migration, invasion and EMT process. (A) The transfection efficiency of miR-19a-3p mimics was measured by RT-qPCR. B. CCK-8 assay was done to measure the viability of PANC-1 and BxPC-3 cell lines. (C‒D) EdU assay was done to evaluate the proliferation in PANC-1 and BxPC-3 cell lines following the co-transfection of sh-LINC01857#1 with pcDNA3.1/SMOC2. (E‒F) The migratory ability of PANC-1 and BxPC-3 cell lines was measured by wound healing assay. (G‒H) Cell invasion in PDAC was measured by Transwell assay. (I) The protein levels of E-cadherin, N-cadherin and vimentin in PANC-1 and BxPC-3 cell lines were analyzed by western blot. *p < 0.05, ^⁎⁎^*p <* 0.01, ^⁎⁎⁎^*p <* 0.001.

Furthermore, the authors aimed to explore whether LINC01857 exerts its biological functions on malignant phenotypes of PDAC cells through SMOC2. Compared with the control group, SMOC2 expression was raised by pcDNA3.1/SMOC2 (Fig. 6A). Further, CCK-8 and EdU assays suggested that pcDNA3.1/SMOC2 partially rescued the suppressive influence of LINC01857 downregulation on the viability and proliferation of PANC-1 and BxPC-3 cells (Fig. 6B‒D). In addition, the wound healing assay result showed that the scratch healing ability of PANC-1 cells and BxPC-3 cell lines was weakened by sh-LINC01857#1, and such effect was neutralized by co-transfection with pcDNA3.1/SMOC2 (Fig. 6E‒F). Also, the Transwell assay revealed that the inhibitive influence of sh-LINC01857#1 on the number of invaded cells was offset by SMO2 overexpression (Fig. 6G‒H). Further, the inhibitory impact of sh-LINC01857#1 on the levels of EMT-relevant protein (N-cadherin and vimentin) was partially reversed by SMOC2 upregulation, and the promotive effect of sh-LINC01857#1 on E-cadherin was also reversed (Fig. 6I). In summary, these results demonstrated that SMOC2 upregulation neutralized the suppressive influence of LINC01857 knockdown on cell proliferation, migration, and invasion.

## Discussion

Emerging evidence has revealed that lncRNAs exert biological functions on the regulation of cellular processes.^24^, ^25^, ^26^ LncRNAs biologically influence cancer progression and metastasis.^27^^,^^28^ In addition, lncRNAs were suggested to be crucial elements in pancreatic cancer, and they have been confirmed to participate in some stages of PDAC progression. In the present study, LINC01857 expression was significantly elevated in PDAC cells and tissues. Downregulation of LINC01857 repressed cell proliferation and motions in PDAC. Additionally, LINC01857 was mainly located in the cytoplasm, suggesting that LINC01857 functions at the post-transcriptional level. Therefore, LINC01857 can play an oncogenic roll in controlling PDAC development by downregulating its expression.

The interaction between lncRNA and miRNAs has been a hot area of research.^29^^,^^30^ Emerging studies have proposed that lncRNAs can modulate mRNA levels by acting as miRNA sponges, which are also identified as competing for endogenous RNAs (ceRNAs)^31^, ^32^, ^33^, ^34^ MicroRNAs (miRNAs) are small endogenous non-coding RNAs. They are confirmed to be specificity elements in post-transcriptional gene silencing.^35^ Additionally, miRNAs have been suggested to be tumor eliminators or cancer-promoting factors in the regulation of cancer progression.^35^, ^36^, ^37^ It has been revealed that the aberrant expression of some miRNAs in PDAC is closely related to cancer development. In detail, it was reported that miR-138-5p inhibits autophagy in PDAC through Sirtuin 1 (SIRT1).^38^ MiR-135 encourages the adaptation of pancreatic cancer cells to metabolic stress by interacting with phosphofructokinase 1.^39^ Notably, the effect of miR-19a-3p differs in different tumors. Studies have found that miR-19a-3p silencing increases osteosarcoma cell's chemosensitivity by upregulating the levels of tumor inhibitor Phosphatase and Tensin homolog (PTEN).^40^ Herein, the authors verified that miR-19a-3p was a downstream target of LINC01857. MiR-19a-3p levels were markedly downregulated in PDAC cells and negatively correlated with LINC01857 levels, indicating that LINC01857 may accelerate cell growth in PDAC by interacting with miR-19a-3p.

Secreted Protein Acidic and Cysteine-rich (SPARC) related Modular Calcium-binding 2 (SMOC2) belongs to the SPARC family, which are markedly upregulated in the process of embryogenesis and wound healing.^19^ Studies have shown that its levels in cancers are markedly raised.^20^^,^^41^ Here, the authors found that SMOC2 was also overexpressed in PDAC and was a potential target for miR-19a-3p. Additionally, it was revealed that the SMOC2 levels had a negative correlation with that of miR-19a-3p while positively correlated with LINC01857 expression. Importantly, SMOC2 overexpression reversed the suppressive influence of LINC01857 downregulation on cell proliferation, migration, and invasion, indicating that LINC01857 influences PDAC development by upregulating SMOC2.

However, some limitations in this study are worth mentioning. *In vivo* assays are required to further validate the findings of the present study. Additionally, the authors will study the expression of LINC01857 in the serum of pancreatic cancer patients and investigate whether the serum expression level of LINC01857 can be used to distinguish between chemoresistant patients and chemosensitive patients with PDAC in the future.

In conclusion, lncRNA LINC01857 facilitates the proliferation, migration, invasion and EMT process in PDAC cells by modulating the miR-19a-3p/SMOC2 axis. The present study provides new perspectives on the investigation of dysregulated genes in PDAC and suggests that the LINC01857/miR-19a-3p/SMOC2 axis might be a possible therapeutic target for patients with PDAC.

## Authors' contributions

Yeting Lu, Dongjian Ying and Shuo Han were responsible for the conceptualization, formal analysis, and project administration of this study. Yeting Lu, Dongjian Ying, Yuan Tian, Yi Ruan, Gong Cheng, Kaiji Lv, Xinhua Zhou and Shuo Han were in cherge of data curation, investigation, methodology, resources, software, supervision, validation, and visualization of the experiments. Yeting Lu and Dongjian Ying are responsible for original draft editing. The final draft was reviwed and edited by all authors.

## Conflicts of interest

The authors declare no conflicts of interest.
